# Health Information Seeking and Behavior in the Korean Population During the COVID-19 Pandemic

**DOI:** 10.3390/healthcare13192539

**Published:** 2025-10-08

**Authors:** Hanna Choi, Meiling Jin, Byungsun Park

**Affiliations:** 1 Department of Nursing Science, Nambu University, Gwang-ju 62271, Republic of Korea; 2 Department of Social Welfare, Gangneung-Wonju National University, Wonju-si 26403, Republic of Korea

**Keywords:** E-health, consumer health information, COVID-19, behavior

## Abstract

**Background:** Online health information seeking emerged as a critical form of public health behavior during the COVID-19 pandemic, generating substantial research interest. However, empirical studies examining health information-seeking patterns among Korean populations and their behavioral outcomes during the pandemic remain limited. Grounded in the information–motivation–behavior skills model, this study investigates online health information-seeking behaviors, including information sources, search terms, and engagement patterns, while also exploring their association with actual health behaviors during the COVID-19 pandemic. **Methods:** A structured survey was developed based on 1014 adults aged 19 years or older using the 2021 Korean version of the Health Information National Trends Survey (K-HINTS) to obtain nationally representative data. We adopted a structural equation model and analyzed the data using SPSS 25.0 and the WordArt site. **Results:** Of the respondents, 74.2% sought health information online, with vaccine details being the most widely searched topic. Mobile phones were the most commonly used devices (75.8%), and 98% searched for health information online via mobile devices at least once a week. Information (β = 0.230, *p* < 0.001), motivation (β = 0.117, *p* < 0.01), and behavior skills (β = 0.117, *p* < 0.01) positively influenced consumers’ behavioral changes regarding health. Behavioral skills also mediated the influences that information seeking and motivation had on behavioral changes. **Conclusions:** This study examines four aspects of online health information seeking through nationally representative COVID-19 data in South Korea. Exploring the relationship between information-seeking and actual health behaviors provides crucial insights for predicting post-pandemic consumer behavior and developing effective public health communication strategies for future crises.

## 1. Background

During the 2019 coronavirus pandemic, consumers in many countries faced unprecedented restrictions in their daily lives [1], while simultaneously experiencing significant barriers to traditional face-to-face healthcare services [2,3]. The initial outbreak in Wuhan, China, and its subsequent global spread prompted widespread online information-seeking behavior as people turned to the Internet for health-related guidance [4]. While health information-seeking behaviors have somewhat decreased since the peak pandemic period, understanding digital health-seeking behaviors remains critically important for ongoing public health preparedness [5]. During the pandemic, online health information seeking became important, as it was the only way to obtain health information [6]; 53.1% of adults reported accessing online health information [7].

Health information-seeking behavior is the act of obtaining information on topics such as health problems, disease information, health risk factors, and health promotion [8]. A systematic review and survey study revealed that during the COVID-19 pandemic, consumers recognized online health information-seeking behavior [9,10]. In particular, information technology and social media have developed rapidly over the past five years, and online health information seeking has gradually attracted consumers’ attention [9]. Consumers believe that online health information-seeking behavior impacts their family and friends when making decisions regarding health treatment, their overall health maintenance approach, and their thoughts about health-related issues [9,11].

With the increased interest in seeking health information, related studies have proliferated, but few have focused on how health information seeking actually affects behaviors towards health [12]. These fact-finding surveys have collected data on a one-time basis, except for China [13] and the United States [14], where the Health Information National Trend Survey (HINTS) is conducted periodically. In addition, only some studies have generalizable results, as they do not investigate specific groups and are not based on a theoretical framework.

Therefore, in this study, we examine how information affects behavioral change based on the information–motivation–behavior skills (IMB) model with nationally representative data from the Korean version of the Health Information National Trend Survey (K-HINTS). The IMB model proposes that behavioral change is facilitated when an individual acquires sufficient information and is motivated to make behavioral changes, which results in improved behavioral skills. The IMB model refers to the information, motivation, and behavioral skills necessary for behavioral change. Information pertains to knowledge regarding health behaviors obtained from various sources. Motivation refers to the attitudes, beliefs, and values influencing individuals and the social support needed to participate in behavioral changes. Behavioral skills encompass practical abilities, such as communication and problem-solving, which are essential for adopting behavioral changes. In this study, we assess information through the concept of access, evaluate motivation factors based on social support, and measure behavioral skills based on health information technology self-efficacy. We assess behavioral changes based on health-related goal management, health-related decision making, and health communication with healthcare providers [15,16].

A study by Tjahjadi [17] conducted among Asian individuals investigated the effects of information and motivation on behavioral changes related to the COVID-19 pandemic in Indonesia. The results showed that information and motivation had a positive impact on consumers’ behavioral changes. Moreover, behavioral skills mediated the effect of information and motivation on behavioral change [18]. In China, information, motivation, behavioral skills, and positive perception of interventions had positive impacts on health behaviors during the COVID-19 pandemic; behavioral skills were the most influential factor on health behavior [18]. In Korea, an examination of consumer experiences related to health information during the COVID-19 pandemic showed that more health-related searches were conducted on the Internet during the COVID-19 pandemic than previously [19]. However, no overall quantitative research data are available on the generalizable health information search status.

This study aims to analyze the data collected in three strata (region, gender, and age) and generalize the survey results based on the IMB theoretical framework. The main research questions are as follows:

1. Where (information source), what (search term), and how (device and frequency) did consumers obtain health-related information during the COVID-19 period?
2. How did the search for health information found in this way affect actual health behavior?
3. By verifying these research questions, we intend to identify consumers’ health information search behavior due to the COVID-19 pandemic and use it as basic data for predicting their post-pandemic behavior.

## 2. Methods

### 2.1. Research Design

This study takes the form of investigative research, that is, a secondary quantitative analysis of 2021 K-HINTS data, to determine information sources, search terms, devices, and the frequency of health information searches during the COVID-19 pandemic, and whether this information, motivation, and behavior resulted in actual behavioral changes.

### 2.2. Data Collection

This study is based on a secondary quantitative analysis of primary online survey data. Data collection for this study was conducted from June 2021 to August 2021. Three-level stratified random sampling was employed to enhance the study’s relevance. The sample size of 1000 individuals was allocated according to the population census by region, gender, and age. Initially, 10 times the number of 1000 respondents were sampled. From this, a subset equivalent to 10 times 500 respondents was extracted. Subsequently, from a master sample panel of 430,000 individuals maintained by Korean Research, 5000 individuals were sampled by region, gender, and age to conduct an online survey via email. For quotas that were not completed, additional emails were sent from the master sample panel to recruit the correct number of individuals to fulfill the constructed quota table (Supplementary Table S1).

South Korea comprises 17 administrative districts. To ensure a wide diversity among potential respondents, a population census from these 17 administrative districts was utilized. The distribution of data from the 17 administrative districts in South Korea is as follows: Gyeonggi-do, 267 (26.0%); Seoul, 192 (18.7%); Busan, 69 (6.7%); Gyeongsangnam-do, 68 (6.6%); Incheon, 59 (5.7%); Gyeongsangbuk-do, 51 (5.0%); Daegu, 49 (4.8%); Chungcheongnam-do, 39 (3.8%); Jeollanam-do, 37 (3.6%); Jeollabuk-do, 35 (3.4%); Chungcheongbuk-do, 33 (3.2%); Gwangju, 29 (2.8%); Gangwon-do, 29 (2.8%); Daejeon, 28 (2.7%); Ulsan, 22 (2.1%); Jeju-do, 12 (1.2%); and Sejong, 9 (0.9%). Detailed information about the online survey process is included in the Checklist for Reporting Results of Internet E-Surveys (CHERRIES) (Supplementary Table S2).

The notice contained the name of the survey, period of participation, expected reward, and expected time required to participate in the survey. We selected the method for completing the survey after all participants who fit the allotted quota had responded. In this way, we collected responses from 1028 adults and, after excluding 14 cases with substantial item nonresponse, retained 1014 cases for the final analysis.

### 2.3. Measures

In this study, a questionnaire was constructed based on the HINTS, and verification was conducted by experts to ensure its correct translation into Korean and the content validity of the questions. Specifically, four experts (one professor from a nursing school, two professors from a medical school, and one experienced tool developer majoring in nursing) translated each question into Korean and verified content validity.

### 2.4. Information Sources

Participants were asked to answer a question with multiple responses about where they had found health- or disease-related information over the past year. The categories included books; brochures or pamphlets; magazines; newspapers; inquiries by phone; the Internet; medical institutions (hospitals, clinics, public health centers, etc.); libraries; family members; friends or colleagues; doctors, nurses, or healthcare providers (dentists, oriental doctors, pharmacists, etc.); non-medical persons (persons involved in complementary and alternative therapy [non-medical persons excluding acquaintances or medical personnel], etc.); TV; and radio. All measurements are included in Supplementary Table S3.

### 2.5. Search Terms

In this study, participants were asked to respond to a subjective question about the type of COVID-19-related health information that they had searched for. The range of responses had no limit, and participants were allowed to freely describe their searches.

### 2.6. Device and Frequency

In this study, participants were asked how many times they had accessed the Internet per week on mobile devices (mobile phones, smartphones, or tablets), home computers (including laptops), work computers (including laptops), school computers (including laptops), and computers (including laptops) in public places (libraries, community offices, etc.). The response categories were every day, 4–6 times a week, 1–3 times a week, less than once a week, and not applicable.

### 2.7. Information–Motivation–Behavioral Skills (IMB) Model

To measure information, motivation, and behavioral skills, we used the existing HINTS questions [14]. The specific questions are given as follows. We measured information with the item “Due to COVID-19, I have been looking for health information online more frequently than before” and motivation with the item “Family, friends, and acquaintances influence my health management.” Measurements were conducted on a 4-point Likert scale ranging from 1 (not at all) to 4 (very much). A higher score indicated a greater influence of information and motivation, and a lower score indicated a lower influence.

In this study, behavioral skills refer to procedural and communicative information that could only be obtained through the evaluation and use of online health information (i.e., a form of digital health information self-efficacy), rather than using the Internet for mere technological convenience. We measured behavioral skills with four items: “Looking for health information and managing health through the Internet using information technology takes less time than using other media;” “Searching for health information and managing health through the Internet using information technology can save effort compared to using other media;” “It is easy to find health information and learn how to manage health through the Internet using information technology;” and “It is convenient to find health information and manage health through the Internet using information technology.” We measured these items on a 4-point Likert scale ranging from 1 (strongly disagree) to 4 (strongly agree). In the structural equation model, we configured the latent variables for behavioral skills with input based on the four questions.

We measured behavioral changes using three questions: “Did your mobile or tablet device help you monitor the progress of your health-related goals?” (e.g., smoking cessation, weight management, diet control, etc.), “Did your mobile or tablet device help you decide how to manage your health or treat diseases?” and “Did your mobile or tablet device help you consult with your medical staff?” with responses of yes (1) or no (0).

### 2.8. Data Analysis

We analyzed the data using IBM SPSS Statistics, version 28 (IBM Corp., Armonk, NY, USA) to identify the participants’ demographic characteristics and information-seeking status. Next, we analyzed the words study subjects used to search for information during the COVID-19 period and conducted an analysis to show specific and empirical evidence for those keywords. The analysis process was conducted as follows. The following text purification process was performed on 1470 words reported by research subjects to be examples of keywords used to search COVID-19-related information.

First, to refine the text data, we excluded words unrelated to COVID-19. Second, we reviewed similar words and synonyms and unified them into one word. Third, we refined the words by reviewing singular and plural forms, spacing, and other variations. Then, we visualized and presented these refined words using the WordArt site [20]. While coding and refining these texts, the researchers conducted cross-validation analysis to increase the validity of the qualitative analysis results.

In line with the information–motivation–behavioral skills (IMB) framework, we employed structural equation modeling (SEM) to assess the effects of information and motivation on behavioral skills and changes in information-seeking behavior. SEM is well-suited for evaluating the joint influence of independent, mediating, and dependent variables within a single theoretically specified model and testing mediation relationships systematically [21]. To examine mediation, we used a bias-corrected percentile bootstrap (5000 resamples; 95% CIs). This nonparametric approach yields more accurate confidence-interval coverage and greater power than the Sobel test, thereby strengthening the validity of our inferences regarding mediation.

For the analysis of the research model, we used the full information maximum likelihood (FIML) estimator, and the model’s goodness of fit was verified through Root Mean Square Error of Approximation (RMSEA ≤ 0.08), the Tucker–Lewis index (TLI ≥ 0.90), and the Comparative Fit Index (CFI ≥ 0.90) [21]. To analyze the data, we used the programs SPSS 25.0 and AMOS 24.0.

## 3. Results

### 3.1. General Characteristics

The participants’ general characteristics are presented in Table 1. Specifically, 515 (50.8%) were men and 499 (49.2%) were women, with an average age of 45.0 (SD = 13.6) years. In terms of education, 166 (16.4%) graduated from high school, and 116 (11.4%) graduated from graduate school or higher. Most participants (649 or 64.0%) were married; 309 (30.5%) were unmarried. In terms of household income, 468 (46.2%) had an income of KRW 2–5 million, and 350 (34.5%) had an income of KRW 5–10 million.

### 3.2. Health Information-Seeking Behaviors

#### 3.2.1. Information Source

**Figure 1:**
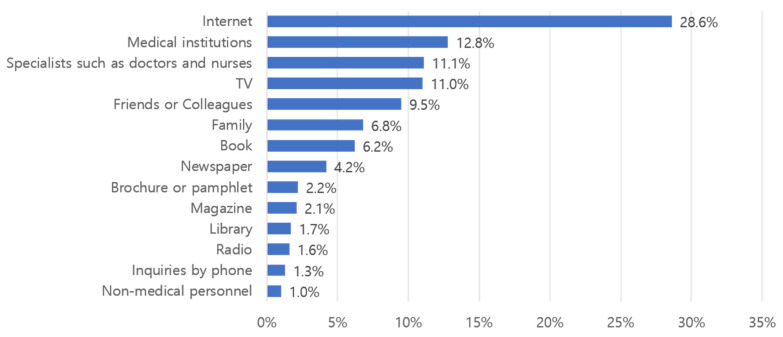
Health-related information-seeking route over the past year (multiple responses) (*N* = 3198).

**Figure 2:**
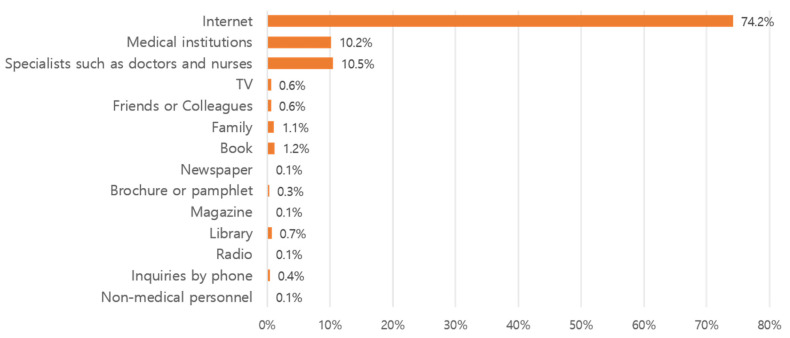
The first method used to search for health-related information (*N* = 1014).

The main results are shown in Figure 1. Regarding sources of information, out of 3198 responses, 916 (28.6%) related to Internet use; 409 (12.8%) indicated medical institutions, and 354 (11.1%) indicated doctors, nurses, or health-related experts. TV was mentioned in 351 (11.0%) cases. Figure 2 shows the results of responses where participants were asked to select the first source they relied on when seeking health- or disease-related information. Of the 1014 respondents, 752 (74.2%) selected the Internet, followed by doctors, nurses, or health-related experts in 106 responses (10.5%) and medical institutions in 103 responses (10.2%).

#### 3.2.2. Search Terms

**Figure 3:**
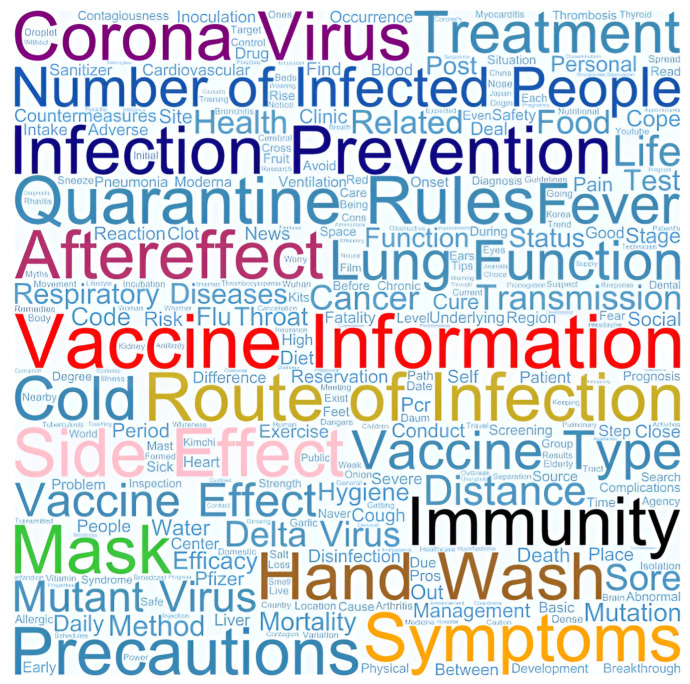
Information researched during the COVID-19 pandemic.

When participants searched for information related to COVID-19, “vaccine information” was searched for 277 times out of 1470 words; “symptoms” was searched 179 times; “coronavirus” was searched 177 times; “infection prevention” was searched 126 times; “side effects” was searched 84 times; “long COVID” was searched 64 times; “mask” was searched 39 times; “number of infected people” was searched 34 times; “hand wash” was searched 28 times; “route of infection” was searched 21 times; and “quarantine rules” was searched 20 times. In addition, participants searched for information on “lung function,” “vaccine effect,” “vaccine type,” “treatment,” “respiratory diseases,” “mutant virus,” “delta virus,” “fever,” and “cancer” (Figure 3).

#### 3.2.3. Device and Frequency

**Figure 4:**
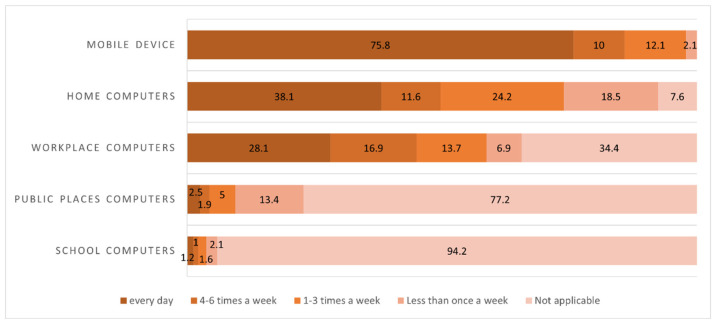
Frequency of online health information seeking using technologies (*N* = 1014).

The results of examining the methods used by participants to search for online health information, as well as the frequency with which these searches were conducted per week, are shown in Figure 4. First, mobile devices had the highest rate of weekly use, with 769 (75.8%) participants using these devices every day, followed by 386 (38.1%) responses for home computers and 285 (28.1%) responses for work computers. Regarding the frequency of device use to search for health information, 769 (75.8%) participants used mobile devices every day, and around 98% conducted mobile phone searches more than once a week. The main apps used were Samsung Health 364 (47.5%), Cashwalk 45 (5.7%), and LG health 26 (3.4%).

A total of 386 (38.1%) participants used home computers daily; 245 (24.2%) participants used them 1−3 times a week; 118 (11.6%) participants used them 4−6 times a week, and 77 (18.5%) participants used them less than once a week. Finally, 285 (28.1%) participants used work computers daily, and 171 (16.9%) used them less than once a week.

### 3.3. Information–Motivation–Behavioral Skills (IMB) Model

Table 2 presents the Pearson product–moment correlations among the main variables—information, motivation, behavioral skills, and behavioral changes—along with descriptive frequencies. All bivariate associations were statistically significant and ranged from r = 0.131 to 0.236 (two-tailed α = 0.05; *p*-values from the *t*-test for r). Distributional screening indicated acceptable normality: the absolute skewness and kurtosis of the main variables did not exceed two and seven, respectively, satisfying the recommended thresholds (Table 2) [22].

### 3.4. Structural Equation Modeling Analysis

We conducted an analysis of the measurement model for the main variables of information, motivation, behavioral skills, and behavioral changes, and the results are presented in Table 3. We found each factor loading of the measurement model to be significant at the level *p* < 0.001; the measurement model’s goodness of fit was χ^2^ = 41.814, df = 23, *p* = 0.010, TLI = 0.989, CFI = 0.993, and RMSEA = 0.028, which is within the acceptable range and suggests that the measurement model fits the data.

**Figure 5:**
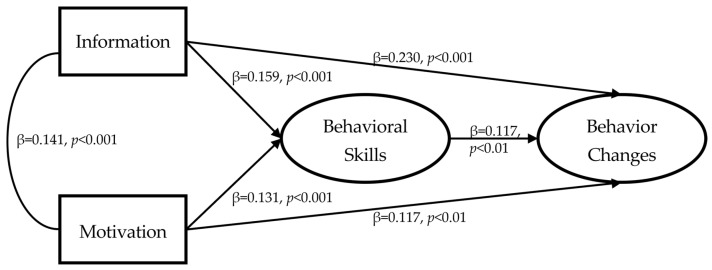
Path coefficient between variables (standardized coefficient).

The results of analyzing the research model used in this study are presented in Figure 5 and Table 4. The research model’s overall fit was excellent, with χ^2^ = 41.814; df = 23; *p* = 0.010; TLI = 0.989; CFI = 0.993; and RMSEA = 0.028. In terms of the path coefficients between the major variables, the higher the value for information, the higher the values for behavioral skills (β = 0.159, *p* < 0.001) and behavioral changes (β = 0.230, *p* < 0.001). Behavioral skills (β = 0.131, *p* < 0.001) and behavioral changes (β = 0.117, *p* < 0.01) also increased. In addition, the higher the value for behavioral skills, the more significant the level of behavioral changes (β = 0.117, *p* < 0.01).

We conducted a bootstrapping analysis on the mediating effect that behavioral skills have on behavioral changes in motivation and health-related information seeking. The mediating effect of behavioral skills on information-seeking behavioral changes was statistically significant at 0.019 (*p* < 0.01), and its mediating effect on behavioral changes for motivation was also significant at 0.015 (*p* < 0.01).

## 4. Discussion

This study examined consumers’ online health information-seeking behavior during the COVID-19 pandemic in South Korea, where the number of Internet users is high, with 93% of the population aged 3 or older using the Internet [23]. Additionally, a recent systematic review confirmed that internet health information seeking increased before and after the COVID-19 pandemic, with search engines, social media, and news portals identified as primary information sources. These findings support the relevance and necessity of this study’s focus [24]. Specifically, we analyzed the structural model for the effect of information seeking—including the type of information sought, where information was obtained, how, and why—on changes in actual health behavior. These results provide basic data to aid future post-pandemic predictions of consumers’ behavior.

The study found that the Internet was the most common information-seeking resource during the COVID-19 period in Korea, at 28.6%. This is similar to the results of a health information-seeking behavior survey conducted on 520 adults in the Southern United States [25]. The Internet has also been confirmed as an important source of health information in China [2]. The results of a study investigating COVID-19-related health information-seeking behavior among Iranian adults aged 19–29 showed that 75.2% started searching for health information on search engines and virtual social media [26]. Search engines are also the preferred source for German university students in Europe with regard to web-based health and COVID-19-related information [27]. This indicates that the Internet was used as the main source of health information during the COVID-19 period, not only in Korea but also worldwide. Among Internet resources, search engines were the preferred mode of searching [28]. The growing importance of online health information-seeking behavior is further supported by systematic review evidence, which indicates a steady increase in research focusing on this phenomenon, establishing it as a critical area of scientific inquiry [29].

The most frequent search term in South Korea was “vaccines,” which was searched 277 times. Next, “vaccine side effects” were of particular interest and searched 84 times. Moreover, as the government’s distancing policy changed according to the aftermath of the COVID-19 pandemic based on the number of confirmed cases, route of infection, and changing quarantine rules, phrases such as “how to prevent COVID-19,” “wearing a mask,” and “washing hands” were ranked at the top among search terms. In contrast, in Germany, the most frequently searched terms for university students were “current spread of COVID-19” (89.6%), “restrictions” (85.9%), “current situation assessments and recommendations of confirmed cases” (77.8%), and “symptoms of COVID-19” (71.5%) [27]. The most retrieved information was on accessing medical treatment, managing self-quarantine, and offline and online support [2]. Thus, during the COVID-19 pandemic, the keywords in consumers’ searches for health information were influenced by the government’s quarantine policy in each country.

Regarding the use rate of devices in online health information seeking, 769 (75.8%) participants used mobile devices daily, and around 98% used mobile devices at least once a week to obtain online health information. A study by Kubb and Foran [28] found that daily Internet users access the Internet via mobile phones, consistent with the finding that smartphones have overtaken desktop computers as the most used device for searching health information [28]. Regarding the frequency of online health information-seeking behavior, more than 60% of health information consumers reported conducting searches at least weekly [9]. Korea has a high rate of daily Internet use at 75.8%, which confirms the high dependence on mobile devices.

This study shows that information, motivation, and behavioral skills have a positive effect on behavioral changes. Health information seeking had a positive effect on attitudes toward COVID-19 vaccination and vaccination intentions [30]. Similarly, a large-scale study of 7218 participants in China also reported that online health information seeking contributed to increased COVID-19 vaccination rates by enhancing understanding of vaccine benefits and improving vaccination acceptance [31]. This suggests that online health information seeking can lead to actual changes in health behavior. Furthermore, according to Jalilian et al. [26], 94.2% of adults in Iran aged 19–29 reported behavioral improvement after obtaining COVID-19-related health information [26].

The study of motivation, which investigated the influence of family, friends, or acquaintances on healthcare, also indicated that the three factors prompting online health information-seeking behavior were personal, family, and friend/peer sickness [9]. The reasons behind Chinese college students’ motivation in seeking COVID-19-related health information were similar: to gain personal protection, obtain the latest information, and obtain information for family and friends. However, changes in health behavior have not been investigated in practice [32].

In this study, the mediating effect of behavioral skills on the ability of information to affect behavioral changes was statistically significant, and the ability to influence the motivation behind behavioral changes was also significant. The results regarding behavioral change were the same as those in a preceding study assessing the mediating influence of behavioral skills on behavioral changes regarding information retrieval and motivation [17]. Compared to countries with widespread access to information and communication technologies (ICT), individuals in countries with low ICT development believed that the Internet is a reliable source of health information or perceived themselves as being able to find the health information they needed online. They were also more likely to find information in this way [33]. Therefore, to increase health equity through changes in individual health behavior, it seems necessary to develop interventions that increase behavioral skills that aid individual behavioral changes.

The limitations of this study are given as follows. First, to maintain focus on theory-specific relations among the core IMB constructs, we excluded demographic covariates (e.g., age, sex, education) and did not conduct multi-group invariance testing. This choice leaves potential confounding and group-wise measurement/structural equivalence untested, which may limit the generalizability and interpretability of the path estimates. Future work should incorporate covariate-adjusted models and formally evaluate configural–metric–scalar invariance across key subgroups. Second, information and motivation were assessed with single items, precluding internal-consistency estimation and potentially narrowing construct coverage. Although these items align with the IMB framework and prior HINTS-based applications, subsequent studies should employ validated multi-item scales (or psychometrically evaluated short forms) and, where appropriate, use fixed-reliability single-indicator latent specifications as part of sensitivity analysis. In line with these limitations, we interpret the findings as distinguishing theory-informed associations among proxy measures of the IMB constructs rather than a definitive test of the full theory.

## 5. Conclusions

This study highlights key trends in consumers’ online health information-seeking behavior during the COVID-19 pandemic. It identifies information sources, search terms, devices, and the frequency at which information was obtained, as well as identifying the effects of information, motivation, and behavioral skills on behavioral changes. Most participants heavily relied on the Internet as a source of health information, particularly for vaccine-related topics. Mobile devices were the predominant means of information retrieval, with a high weekly search rate. Importantly, this study revealed positive associations between information, motivation, and behavioral skills and changes in consumers’ health behavior, with behavioral skills acting as a mediator for the impact of information and motivation on behavioral change. This study’s findings will significantly contribute to identifying consumers’ health information-seeking behavior due to the pandemic and predict post-pandemic behavior. By applying lessons from this period, future preparedness can better meet public needs through timely, accurate information, leading to more informed health behaviors.

## Figures and Tables

**Table 1:** General characteristics of participants (*N* = 1014).

Characteristics	Participants
Age (years), mean (SD)	45.0 (13.6)
Age (years), *n* (%)	
19–39	368 (36.3)
40–59	452 (44.6)
>60	194 (19.1)
Gender, *n* (%)	
Male	515 (50.8)
Female	499 (49.2)
Education, *n* (%)	
Elementary and middle school graduate	10 (1.0)
High school graduate	166 (16.4)
University/college student	51 (5.0)
University/College graduate	652 (64.3)
Graduate school student	19 (1.9)
Grad school graduate and above	116 (11.4)
Marital status, *n* (%)	
Single	309 (30.5)
Married	649 (64.0)
Divorced	35 (3.5)
Widowed	17 (1.7)
Separated and cohabiting	4 (0.4)
Family income, *n* (%)	
<KRW 1 million	43 (4.2)
KRW 1 million~KRW 2 million	77 (7.6)
KRW 2 million~KRW 5 million	468 (46.2)
KRW 5 million~KRW 10 million	350 (34.5)
<KRW 10 million	76 (7.5)

**Table 2:** Correlations and descriptive statistics of variables (*N* = 1014).

Variable	Information	Motivation	Behavioral Skills	Behavior Changes
Information				
*r*	1	0.141	0.170	0.236
*p* value	-	<0.001	<0.001	<0.001
Motivation				
*r*	0.141	1	0.143	0.131
*p* value	<0.001	-	<0.001	<0.001
Behavioral Skills				
*r*	0.170	0.143	1	0.138
*p* value	<0.001	<0.001	-	<0.001
Behavior Changes				
*r*	0.236	0.131	0.138	1
*p* value	<0.001	<0.001	<0.001	-
Mean	2.79	2.91	12.7130	1.6036
Std. Deviation	0.707	0.571	1.87557	1.11378
Skewness	−0.104	−0.326	0.098	−0.122
Kurtosis	−0.272	0.905	0.609	−1.339
Minimum	1	1	4	0
Maximum	4	4	16	3

**Table 3:** Factor loading and correlation coefficient of variables (*N* = 1014).

Variable		Mean	S.E.		C.R
Information		2.794	0.022		125.890 ^a^
Motivation		2.906	0.018		162.123 ^a^
		B	β	S.E.	C.R
Behavioral Skills	item 1	1.000	0.815	-	-
	item 2	0.986	0.852	0.033	29.455 ^a^
	item 3	0.942	0.744	0.037	25.170 ^a^
	item 4	0.920	0.801	0.033	27.563 ^a^
Behavior Changes	item 1	1.000	0.485	-	-
	item 2	1.593	0.769	0.161	9.916 ^a^
	item 3	1.129	0.558	0.106	10.631 ^a^
Correlation		B	β	S.E.	C.R
Information ↔ Motivation		0.057	0.141	0.013	4.428 ^a^
Information ↔ Behavioral Skills		0.057	0.178	0.011	5.217 ^a^
Information ↔ Behavior Changes		0.045	0.267	0.007	6.071 ^a^
Motivation ↔ Behavioral Skills		0.040	0.154	0.009	4.536 ^a^
Motivation ↔ Behavior Changes		0.023	0.167	0.006	4.146 ^a^
Behavioral Skills ↔ Behavior Changes		0.019	0.176	0.005	4.094 ^a^
Model fit index	χ^2^ = 41.814, df = 23, *p* = 0.010, TLI = 0.989, CFI = 0.993, RMSEA = 0.028				

^a^ *p* < 0.001. B = Unstandardized coefficient; β = Standardized coefficient; S.E. = Standard Error; C.R. = Critical Ratio.

**Table 4:** Path coefficients between factors in the study model (*N* = 1014).

Path		B	β	S.E.	C.R.
Information → Behavioral Skills		0.103	0.159	0.021	4.802 ^b^
Information → Behavioral Changes		0.078	0.230	0.014	5.537 ^b^
Motivation → Behavioral Skills		0.105	0.131	0.027	3.968 ^b^
Motivation → Behavioral Changes		0.049	0.117	0.016	3.051 ^a^
Behavioral Skills → Behavioral Changes		0.062	0.117	0.022	2.860 ^a^
Path		Total effect	Direct effect	Mediated effect	Indirect confidence interval
Information → Behavioral Skills → Behavioral Changes		0.248	0.230	0.019	0.009–0.036 ^a^
Motivation → Behavioral Skills → Behavioral Changes		0.133	0.117	0.015	0.006–0.032 ^a^
Model fit index	χ^2^ = 41.814, df = 23,	*p* = 0.010, TLI = 0.989, CFI = 0.993, RMSEA = 0.028			

^a^ *p* < 0.01, ^b^ *p* < 0.001. B = Unstandardized coefficient; β = Standardized coefficient; S.E. = Standard Error; C.R. = Critical Ratio.

## Data Availability

The raw data supporting the conclusions of this article will be made available by the authors on request.

## Supplementary Materials

The following supporting information can be downloaded at: https://www.mdpi.com/article/10.3390/healthcare13192539/s1, Table S1: Participants based on population census and master sample panel; Table S2: Checklist for Reporting Results of Internet E-Surveys (CHERRIES); Table S3: Instrument’s questions and multiple-choice options on this study.

## Abbreviations

The following abbreviations are used in this manuscript:

IMB	Information–Motivation–Behavior skills
K-HINTS	Korean version of the Health Information National Trend Survey
HINTS	Health Information National Trend Survey
ICT	Information and Communications Technologies
