# Supplementary material for: Health Information Seeking and Behavior in the Korean Population During the COVID-19 Pandemic
Source: Healthcare (Basel). 2025 Oct 8;13(19):2539. doi: 10.3390/healthcare13192539 (PMC12524546; doi:10.3390/healthcare13192539)
Supplement: Supplementary file 1 [file healthcare-13-02539-s001.zip › _Supplementary _Table S1.pdf]

Supplementary Table S1. Participants based on population census and master sample panel

Administrative districts / Age	Sample (Population census)						Master sample panel
	Male			Female			
	19~39	40~59	over 60	19~39	40~59	over 60	
Gyeonggi-do	52 (1,924,795)	62 (2,284,176)	22 (800,596)	49 (1,766,065)	60 (2,219,818)	22 (810,068)	101,807
Seoul	38 (1,410,092)	39 (1,457,683)	16 (590,828)	39 (1,460,403)	42 (1,497,205)	18 (661,431)	124,794
Busan	12 (427,733)	14 (520,120)	7 (255,221)	12 (401,344)	15 (531,468)	9 (285,101)	29,666
Gyeongsangnam-do	12 (408,184)	16 (564,458)	7 (240,072)	9 (347,642)	14 (536,284)	10 (242,968)	18,163
Incheon	12 (410,539)	14 (494,145)	5 (190,465)	10 (379,980)	13 (484,905)	5 (194,609)	28,199
Gyeongsangbuk-do	8 (309,345)	12 (430,020)	6 (211,746)	7 (252,725)	11 (397,776)	7 (213,517)	13,547
Daegu	10 (316,904)	10 (387,866)	4 (159,605)	9 (283,562)	11 (402,020)	5 (176,236)	21,325
Chungcheongnam-do	7 (273,152)	10 (350,549)	4 (147,046)	6 (227,619)	8 (309,326)	4 (146,013)	11,375
Jeollanam-do	7 (204,977)	8 (302,406)	5 (141,305)	5 (172,103)	7 (261,239)	5 (138,442)	8,426
Jeollabuk-do	6 (207,944)	8 (288,184)	4 (130,313)	6 (184,901)	7 (269,163)	4 (133,233)	10,513
Chungcheongbuk-do	6 (210,988)	8 (260,330)	3 (118,388)	5 (176,500)	7 (244,281)	4 (117,512)	9,136
Gwangju	5 (201,106)	6 (232,454)	2 (80,067)	6 (188,097)	7 (236,177)	3 (88,108)	13,202
Gangwon-do	5 (184,631)	7 (247,308)	3 (125,974)	4 (154,140)	6 (233,531)	4 (126,621)	9,115
Daejeon	6 (209,368)	6 (233,363)	2 (90,361)	5 (190,481)	6 (235,332)	3 (96,188)	15,387
Ulsan	4 (157,289)	5 (198,009)	2 (76,524)	4 (130,094)	5 (191,379)	2 (75,515)	8,089
Jeju-do	2 (83,942)	3 (115,729)	1 (40,694)	2 (78,808)	3 (108,164)	1 (41,051)	3,051
Sejong	1 (49,360)	2 (60,384)	1 (16,035)	1 (49,922)	3 (57,142)	1 (16,306)	401
