# Supplementary material for: Health Information Seeking and Behavior in the Korean Population During the COVID-19 Pandemic
Source: Healthcare (Basel). 2025 Oct 8;13(19):2539. doi: 10.3390/healthcare13192539 (PMC12524546; doi:10.3390/healthcare13192539)
Supplement: Supplementary file 1 [file healthcare-13-02539-s001.zip › Supplementary Table S3.pdf]

Supplementary Table S3. Instrument's questions and multiple-choice options on this study

Domain		Response option	Questionnaire item
Information Source		Multiple Choice (Duplicate responses)	The most recent time (In the past 12 months) you looked for information about health or medical topics, where did you go first? Mark all that apply. *① Book ② Brochure or pamphlet ③ Magazine ④ Newspaper ⑤ Inquiries by phone ⑥ Internet ⑦ Medical institutions ⑧ Library ⑨ Family ⑩ Friend or Colleagues ⑪ Specialists such as doctors and nurses ⑫ Non-medical personnel ⑬ TV ⑭ Radio*
Search Term		Write Text	What health information related to covid-19 did you search for?
Device and Frequency		5 multiple choice (Everyday, 4-6 times a week, 1-3 times a week, Less than once a week, Not applicable)	How often do you access the Internet through using home computers, workplace computers, school computers, public places computers (including laptop), and mobile device (phones, smartphones, or tablets)?
IMB Model Variables	Information	4 multiple choice (Strongly agree, agree, disagree, strongly disagree)	COVID-19 has increased the frequency of online health information seeking compared to before.
	Motivation		My family, friends, and acquaintances influence my health management
	Behavioral Skills		Using information technology to find health information and manage health (through the internet) is more time-efficient compared to using other media.
			Using information technology to find health information and manage health (through the internet can reduce effort compared to using other media.
			Learning to use information technology to find health information and manage health (through the internet) is easy.
			Using information technology to find health information and manage health (through the internet) is convenient.
	Behavior Changes	Yes or No	Helped you track progress on a health-related goal such as quitting smoking, losing weight, or increasing physical activity?
			Helped you make a decision about how to treat an illness or condition?
			Helped you in discussions with your health care provider?
