# Supplementary material for: Health Information Seeking and Behavior in the Korean Population During the COVID-19 Pandemic
Source: Healthcare (Basel). 2025 Oct 8;13(19):2539. doi: 10.3390/healthcare13192539 (PMC12524546; doi:10.3390/healthcare13192539)
Supplement: Supplementary file 1 [file healthcare-13-02539-s001.zip › _Supplementary _Table S2.pdf]

Supplementary Table S2. Checklist for Reporting Results of Internet E-Surveys (CHERRIES)

Item Category	Checklist Item	Explanation	Description
Design	Describe survey design	Describe target population, sample frame. Is the sample a convenience sample? (In "open" surveys this is most likely.)	The target population was Korean people aged over 18. A 3-level of stratified random sampling was employed to enhance the study's relevance (i.e., a computer-aided web interview targeting master sample panels of Korea Research Co., Ltd); it involved 1,193,000 nationwide respondents through online surveys.
IRB (Institutional Review Board) approval and informed consent process	IRB approval	Mention whether the study has been approved by an IRB.	This research, as a secondary data analysis study based on the 2021 K-HINTS data, was conducted after approval of a review exemption by the Institutional Review Board of ** University, to which the researcher belongs (*******-2023-HR-012). The 2021 K-HINTS data were also collected after review by the Institutional Review Board of ** University (*******-2021-HR-006).
	Informed consent	Describe the informed consent process. Where were the participants told the length of time of the survey, which data were stored and where and for how long, who the investigator was, and the purpose of the study?	All participants, as members of a web-based panel, had already provided informed consent to participate in online surveys. Informed consent for the present survey was obtained from all those agreeing to complete a survey, with participants informed on the welcome page that the survey concerned health issues, that it would take approximately 25 minutes to complete, that all responses were confidential and anonymous, and that reporting would be on an aggregate level only. Consent was indicated when respondents clicking the "Go to Survey" button from this page.
	Data protection	If any personal information was collected or stored, describe what mechanisms were used to protect unauthorized access.	Korea Research manages panels according to ESOMAR guidelines and ISO/DIS 26362 standards after panel recruitment.<br><br>For specific information on personal data protection, please refer to the following link: https://www.hrc-ms.com/foot/priv.asp
Development and pre-testing	Development and testing	State how the survey was developed, including whether the usability and technical functionality of the electronic	Below are the details of the development server and programming language:

		questionnaire had been tested before fielding the questionnaire.	Web Server: Apache/2.4.53<br>DB Server: MySQL 5.7.11<br>Programming Language: Perl<br>Development Country: South Korea<br>Development Company: Korea Research<br>Initial Development: Since 2006 (ongoing development, modification, and enhancement as of 2024)<br>To check for web vulnerabilities, an external organization conducts annual inspections. Before deploying the questionnaire, the programmed survey underwent comprehensive testing to ensure functionality and user-friendliness.
Recruitment process and description of the sample having access to the questionnaire	Open survey versus closed survey	An “open survey” is a survey open for each visitor of a site, while a closed survey is only open to a sample which the investigator knows (password-protected survey).	Korea Research conducts data collection through its self-built panel, which comprises respondents from across the nation (1,193,000 individuals). The survey method employed is closed-ended, and the website secures web survey items through SSL (Secure Sockets Layers) certification
	Contact mode	Indicate whether or not the initial contact with the potential participants was made on the Internet. (Investigators may also send out questionnaires by mail and allow for Web-based data entry.)	Panel recruitment is conducted through various channels as follows, and after recruitment, data collection follows the process outlined below.<br><br>*Korea Research Panel Recruitment Channels

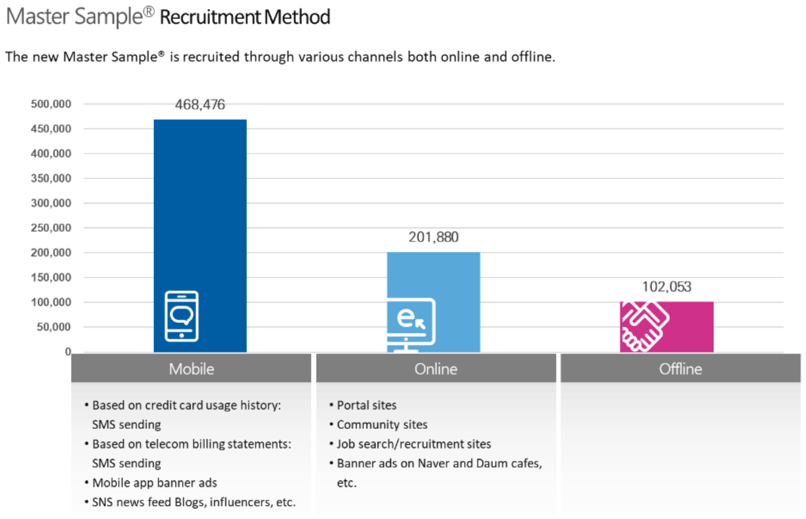

* Data-Collection Process

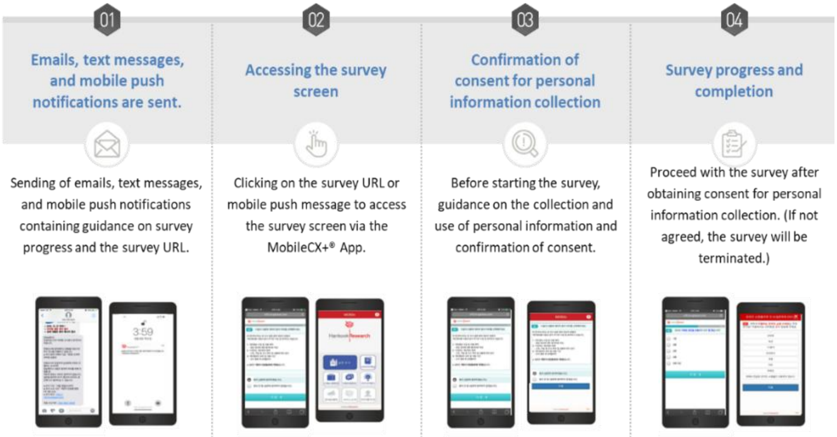

Advertising the survey	How/where was the survey announced or advertised? Some examples are offline media (newspapers), or online (mailing lists – If yes, which ones?) or	Korea Research notifies pre-recruited panelists of surveys via	When the survey message arrives, you can quickly participate via mobile push messages.

		banner ads (Where were these banner ads posted and what did they look like?). It is important to know the wording of the announcement as it will heavily influence who chooses to participate. Ideally the survey announcement should be published as an appendix.	mobile/email. Panelists interested in taking part in the survey click on the web survey link provided in the notification. Below is the initial survey notification screen:<br><br>*** Survey Notification Screen**	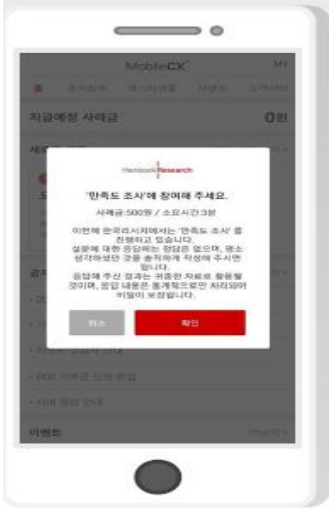
Survey administration	Web/E-mail	State the type of e-survey (eg, one posted on a Web site, or one sent out through e-mail). If it is an e-mail survey, were the responses entered manually into a database, or was there an automatic method for capturing responses?	The type of survey received by the panelists applies to both mobile and email platforms. Data from individuals who participate in surveys via mobile/email are automatically stored in the database.	

* The step-by-step process of Korea Research's web survey is as follows:

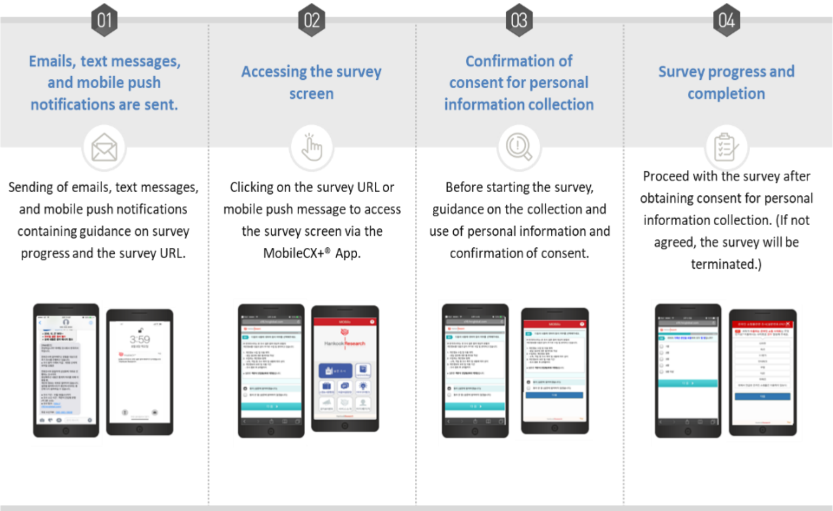

Context	Describe the Web site (for mailing list/newsgroup) in which the survey was posted. What is the Web site about, who is visiting it, what are visitors normally looking for? Discuss to what degree the content of the Web site could pre-select the sample or influence the results. For example, a survey about vaccination on an anti-immunization Web site will have different results from a Web survey conducted on a government Web site	Korea Research conducts web surveys targeting pre-recruited panels via mobile/email. Specifically, for mobile participants, surveys are notified through the MobileCX app developed in-house by Korea Research, whereas email participants receive notifications through their personal email.
Mandatory/voluntary	Was it a mandatory survey to be filled in by every visitor who wanted to enter the Web site, or was it a voluntary survey?	If you wish to participate in the survey, you can take part voluntarily and discontinue the survey at any time.

	Incentives	Were any incentives offered (eg, monetary, prizes, or non-monetary incentives such as an offer to provide the survey results)?	Korea Research provides panel incentives in cash, and the incentive for this survey is 2,500 Korean won. Incentives are paid after the completion of the survey.
	Time/Date	In what timeframe were the data collected?	Surveys were completed between June 2021 to August 2021.
	Randomization of items or questionnaires	To prevent biases items can be randomized or alternated.	Not applicable.
	Adaptive questioning	Use adaptive questioning (certain items, or only conditionally displayed based on responses to other items) to reduce number and complexity of the questions.	Not applicable.
	Number of Items	What was the number of questionnaire items per page? The number of items is an important factor for the completion rate.	The web survey interface follows the principle of displaying one question per page. However, for scale items, detailed subitems are included in the display.
	Number of screens (pages)	Over how many pages was the questionnaire distributed? The number of items is an important factor for the completion rate.	The web survey interface displays one question per page, resulting in the same number of pages as the number of survey items. However, there are two additional pages at the beginning for instructions and at the end to indicate the completion of the survey, adding to the total number of pages.
	Completeness check	It is technically possible to do consistency or completeness checks before the questionnaire is submitted. Was this done, and if "yes", how (usually JAVAScript)? An alternative is to check for completeness after the questionnaire has been submitted (and highlight mandatory items). If this has been done, it should be reported. All	Korea Research conducts real-time inspections through its data-verification system. For instance, it ensures that multiple accesses from the same IP are not allowed and eliminate insincere responses from open-ended questions in advance.

<table>
<tr><td></td><td></td><td>items should provide a non-response option such as "not applicable" or "rather not say", and selection of one response option should be enforced.</td><td></td></tr>
<tr><td></td><td>Review step</td><td>State whether respondents were able to review and change their answers (eg, through a Back button or a Review step which displays a summary of the responses and asks the respondents if they are correct).</td><td>Once respondents move on to the next question after answering, they cannot return to the previous question.</td></tr>
<tr><td rowspan="2">Response rates</td><td>Unique site visitor</td><td>If you provide view rates or participation rates, you need to define how you determined a unique visitor. There are different techniques available, based on IP addresses or cookies or both.</td><td>Responses from the same IP address are automatically excluded for the same survey. Additionally, in the case of the Korea Research Master Panel, each panel has a unique code, which allows for the collection of data based on these distinct codes, enabling the counting of unique visitors.</td></tr>
<tr><td>View rate (Ratio of unique survey visitors/unique site visitors)</td><td>Requires counting unique visitors to the first page of the survey, divided by the number of unique site visitors (not page views!). It is not unusual to have view rates of less than 0.1 % if the survey is voluntary.</td><td>In this survey, the view rate corresponds to the number of opens divided by the number of dispatches. Because the survey is conducted by dispatching notifications rather than direct visits by the panel, the number of dispatches is used instead of the number of unique site visitors. The view rate for this survey is 19.3%.
<table>
<tr><th>NO</th><th>Category</th><th>Total</th></tr>
<tr><td>1</td><td>Number of Dispatches</td><td>19,515</td></tr>
<tr><td>2</td><td>Number of Opens</td><td>3768</td></tr>
<tr><td>3</td><td>Total Number of Participants</td><td>1541</td></tr>
<tr><td>4</td><td>- Number Completed</td><td>1057</td></tr>
<tr><td>5</td><td>- Number Disqualified</td><td>18</td></tr>
<tr><td>6</td><td>- Quota Over</td><td>99</td></tr>
<tr><td>7</td><td>- Survey Discontinued</td><td>338</td></tr>
</table></td></tr>
</table>

<table>
<tr><td></td><td></td><td><table><tr><td>8</td><td>- Number of Insincere Respondents</td><td>29</td></tr></table></td></tr>
<tr><td>Participation rate (Ratio of unique visitors who agreed to participate/unique first survey page visitors)</td><td>Count the unique number of people who filled in the first survey page (or agreed to participate, for example by checking a checkbox), divided by visitors who visit the first page of the survey (or the informed consents page, if present). This can also be called "recruitment" rate.</td><td>In this survey, the participation rate corresponds to the total number of participants divided by the number of opens. The participation rate for this survey is 40.9%.
<table>
<tr><th>NO</th><th>Category</th><th>Total</th></tr>
<tr><td>1</td><td>Number of Dispatches</td><td>19,515</td></tr>
<tr><td>2</td><td>Number of Opens</td><td>3768</td></tr>
<tr><td>3</td><td>Total Number of Participants</td><td>1541</td></tr>
<tr><td>4</td><td>- Number Completed</td><td>1057</td></tr>
<tr><td>5</td><td>- Number Disqualified</td><td>18</td></tr>
<tr><td>6</td><td>- Quota Over</td><td>99</td></tr>
<tr><td>7</td><td>- Survey Discontinued</td><td>338</td></tr>
<tr><td>8</td><td>- Number of Insincere Respondents</td><td>29</td></tr>
</table></td></tr>
<tr><td>Completion rate (Ratio of users who finished the survey/users who agreed to participate)</td><td>The number of people submitting the last questionnaire page, divided by the number of people who agreed to participate (or submitted the first survey page). This is only relevant if there is a separate "informed consent" page or if the survey goes over several pages. This is a measure for attrition. Note that "completion" can involve leaving questionnaire items blank. This is not a measure for how completely questionnaires were filled in. (If you</td><td>In this survey, the completion rate corresponds to the number of completions divided by the total number of participants. The completion rate for this survey is 68.6%.
<table>
<tr><th>NO</th><th>Category</th><th>Total</th></tr>
<tr><td>1</td><td>Number of Dispatches</td><td>19,515</td></tr>
<tr><td>2</td><td>Number of Opens</td><td>3768</td></tr>
<tr><td>3</td><td>Total Number of Participants</td><td>1541</td></tr>
<tr><td>4</td><td>- Number Completed</td><td>1057</td></tr>
<tr><td>5</td><td>- Number Disqualified</td><td>18</td></tr>
<tr><td>6</td><td>- Quota Over</td><td>99</td></tr>
</table></td></tr>
</table>

		need a measure for this, use the word "completeness rate".)	7	- Survey Discontinued	338
			8	- Number of Insincere Respondents	29
Preventing multiple entries from the same individual	Cookies used	Indicate whether cookies were used to assign a unique user identifier to each client computer. If so, mention the page on which the cookie was set and read, and how long the cookie was valid. Were duplicate entries avoided by preventing users access to the survey twice; or were duplicate database entries having the same user ID eliminated before analysis? In the latter case, which entries were kept for analysis (eg, the first entry or the most recent)?	During the data-collection phase, cookies do not play a decisive role in identifying users. The web survey is conducted with the Korea Research Master Panel, which has a unique code for each panel member. Data are collected based on these distinct codes. A panel member can participate only once per survey, and if they attempt to access a survey, they have already completed it, and they will be redirected to a screen indicating that they have already participated in the survey.<br>As a result, cookies are not used, and the collected data are stored in the database based on panel information separately.		
	IP check	Indicate whether the IP address of the client computer was used to identify potential duplicate entries from the same user. If so, mention the period of time for which no two entries from the same IP address were allowed (eg, 24 hours). Were duplicate entries avoided by preventing users with the same IP address access to the survey twice; or were duplicate database entries having the same IP address within a given period of time eliminated before analysis? If the latter, which entries were kept for analysis (eg, the first entry or the most recent)?	For the same survey, responses from the same IP address are excluded in advance. They are automatically classified as fraudulent responses and are excluded beforehand.		

	Log file analysis	Indicate whether other techniques to analyze the log file for identification of multiple entries were used. If so, please describe.	Log files are managed by the unique number of the Master Panel, which is a part of the Korea Research panel. Web surveys are conducted targeting the Korea Research Master Panel, and because each panel member has a distinct unique code, data are collected based on these unique codes. Access records are managed based on the unique number of the Master Panel.
	Registration	In "closed" (non-open) surveys, users need to login first and it is easier to prevent duplicate entries from the same user. Describe how this was done. For example, was the survey never displayed a second time once the user had filled it in, or was the username stored together with the survey results and later eliminated? If the latter, which entries were kept for analysis (eg, the first entry or the most recent)?	When a user attempts to access the survey for the second time after completing it, they will receive a notification indicating that they have already participated in the survey, and they will not be redirected to the survey screen. Therefore, this applies to cases where the survey is not displayed again.
Analysis	Handling of incomplete questionnaires	Were only completed questionnaires analyzed? Were questionnaires which terminated early (where, for example, users did not go through all questionnaire pages) also analyzed?	Uncompleted surveys are excluded from both data collection as complete data and from analysis data, as they correspond to cases where the participant did not agree to participate in the survey.
	Questionnaires submitted with an atypical timestamp	Some investigators may measure the time people needed to fill in a questionnaire and exclude questionnaires that were submitted too soon. Specify the timeframe that was used as a cut-off point, and describe how this point was determined.	The average response time for this survey is 37 minutes. During internal pretesting, it was expected that it would take at least 20 minutes. Respondents who took less than 20 minutes to complete the survey were considered as insincere respondents and were excluded after the survey was completed.
	Statistical correction	Indicate whether any methods such as weighting of items or propensity scores have been used to adjust for the non-	To ensure representativeness, the sampling method utilized the population statistics from the Ministry of Public Administration and Security as of June 2023. The sample was allocated proportionally by

	representative sample; if so, please describe the methods.	region, gender, and age group based on these statistics. Dividing the country into 17 provinces and conducting nationwide data collection are other methods to enhance representativeness by considering geographical diversity.
